# Novel Hypoxia-Related Gene Signature for Risk Stratification and Prognosis in Hepatocellular Carcinoma

**DOI:** 10.3389/fgene.2021.613890

**Published:** 2021-06-14

**Authors:** Quanxiao Li, Limin Jin, Meng Jin

**Affiliations:** ^1^Department of Radiation Oncology, The First Affiliated Hospital of Sun Yat-sen University, Guangzhou, China; ^2^Department of Interventional Oncology, The First Affiliated Hospital of Sun Yat-sen University, Guangzhou, China; ^3^Department of Hepatobiliary and Pancreatic Surgery, The First Hospital of Jilin University, Changchun, China; ^4^Department of Anesthesia, The First Hospital of Jilin University, Changchun, China

**Keywords:** prognosis, hypoxia, hepatocellular carcinoma, international cancer genome consortium, the cancer genome atlas, gene expression omnibus

## Abstract

Hepatocellular carcinoma (HCC) is the most common form of liver cancer with limited therapeutic options and low survival rate. The hypoxic microenvironment plays a vital role in progression, metabolism, and prognosis of malignancies. Therefore, this study aims to develop and validate a hypoxia gene signature for risk stratification and prognosis prediction of HCC patients. The Cancer Genome Atlas (TCGA) and International Cancer Genome Consortium (ICGC) databases were used as a training cohort, and one Gene Expression Omnibus database (GSE14520) was served as an external validation cohort. Our results showed that eight hypoxia-related genes (HRGs) were identified by the least absolute shrinkage and selection operator analysis to develop the hypoxia gene signature and demarcated HCC patients into the high- and low-risk groups. In TCGA, ICGC, and GSE14520 datasets, patients in the high-risk group had worse overall survival outcomes than those in the low-risk group (all log-rank *P* < 0.001). Besides, the risk score derived from the hypoxia gene signature could serve as an independent prognostic factor for HCC patients in the three independent datasets. Finally, a nomogram including the gene signature and tumor-node-metastasis stage was constructed to serve clinical practice. In the present study, a novel hypoxia signature risk model could reflect individual risk classification and provide therapeutic targets for patients with HCC. The prognostic nomogram may help predict individualized survival.

## Introduction

Primary liver cancer is the third most common cause of cancer-related deaths with limited therapeutic options and low survival rate (Sung et al., 2021), among which hepatocellular carcinoma (HCC) is the most common form. Following the developments of diagnostic and therapeutical strategies of HCC, the need for molecular and genetic signature that can predictively reflect the prognosis of patients in advance is constantly growing in clinical practice.

Due to the formation of tumor neovascularization and vigorous tumor metabolism (Brown and Wilson, 2004; Mucaj et al., 2012), a parallel to increase oxygen demand with reduced oxygen supply leads to an oxygen imbalance. Therefore, the presence of hypoxia is a common feature in approximately half of solid tumors (Majmundar et al., 2010; Lee et al., 2017), including HCC (Jain, 2005; Wu et al., 2007). As a vital organ with unique anatomical base, the liver seems insulated to oxygen tensions in the normal condition, while hypoxia often presents during cainogenesis (Wilson et al., 2014). A central regulator of oxygen detection and adaptation at the cellular level, hypoxia-inducible factor (HIF) has been demonstrated to activate genes that control cellular oxygen homeostasis (Choudhry and Harris, 2018), metabolic reprogramming, cell proliferation, invasion and metastasis, apoptosis, and resistance to therapies in various types of cancer (Rankin and Giaccia, 2016). Mechanistically, HIF could bind to specific DNA sequences in target genes in numerous signaling pathways (Wang et al., 1995). However, therapies on hypoxia-related genes (HRGs) are still limited due to lack of evidence and abundant difficulties in evaluating tumor hypoxia.

Recently, the genome-wide expression profiling datasets have been generally applied to discover potential cancer biomarkers *via* single-gene research. So far, few studies have constructed prognostic models based on a combination of multiple HRGs in HCC (Chang et al., 2019; Zhang et al., 2020); however, the performance of the nomogram incorporating hypoxia gene signature and clinical features in predicting survival outcome of HCC patients was not satisfactory in a recent research. Therefore, we aimed to explore a novel HRG-based signature for risk stratification and suggest therapeutic targets in HCC. Furthermore, a nomogram combined with hypoxia gene signature and prognostic clinical risk factors was established to achieve better prognosis prediction of HCC patients in this study.

## Materials and Methods

### Datasets

The mRNA-seq transcriptome profiling and corresponding clinical data of HCC patients were extracted from The Cancer Genome Atlas (TCGA) database (https://portal.gdc.cancer.gov/) and International Cancer Genome Consortium (ICGC) data portal (https://dcc.icgc.org/projects/LIRI-JP), respectively. The gene expression files of the two datasets were merged into one cohort as a training cohort (Huo et al., 2020). Similarly, GSE14520 (GPL3921, Affymetrix HT Human Genome U133A Array) with RNA sequencing and clinical information were downloaded from the Gene Expression Omnibus (GEO) dataset as an external validation cohort (https://www.ncbi.nlm.nih.gov/geo/). Patients with a follow-up period <1 month were excluded. Besides, characteristics of included HCC patients in three independent cohorts are shown in Table 1. The batch effects of RNA sequencing datasets were eliminated using “SVA” R package. Moreover, the hypoxia-related genes (HRG) were obtained from the HALLMARK-HYPOXIA gene set in Gene-Set Enrichment Analysis (GSEA) (https://www.gsea-msigdb.org/gsea/index.jsp).

**Table 1:** Clinical characteristics of the hepatocellular carcinoma patients in three independent cohorts.

**Characteristics**	**TCGA cohort**	**ICGC cohort**	**GSE14520 cohort**
	**(*n =* 343)**	**(*n =* 229)**	**(*n =* 221)**
**Age**			
<60y	157 (45.8)	44 (19.2)	178 (80.5)
≥60y	186 (54.2)	185 (80.8)	43 (19.5)
**Gender**			
Male	233 (67.9)	168 (73.4)	191 (86.4)
Female	110 (32.1)	61 (26.6)	30 (13.6)
**TNM stage**			
I	166 (48.4)	36 (15.7)	93 (42.1)
II	80 (23.3)	105 (45.9)	77 (34.8)
III	94 (27.4)	69 (30.1)	49 (22.2)
IV	3 (0.9)	19 (8.3)	0
**Histologic grade**			
1	53 (15.5)	NA	NA
2	165 (48.1)	NA	NA
3	113 (32.9)	NA	NA
4	12 (3.5)	NA	NA
**AFP**			
≤ 300 ng/ml	198 (57.7)	NA	119 (53.8)
>300 ng/ml	62 (18.1)	NA	100 (45.2)
**Survival status**			
OS years (median)	4.83	2.14	4.35
Alive	220 (64.1)	189 (82.5)	136 (61.5)
Dead	123 (35.9)	40 (17.5)	85 (38.5)

*OS, overall survival*.

### Development and Validation of Prognostic Gene Signature

The relationship between HRGs and the overall survival (OS) of HCC in the training cohort was first detected *via* the univariate Cox regression analysis. The HRGs with a *P* < 0.2 on univariate analysis were selected for further analysis. To reduce overfitting, the least absolute shrinkage and selection operator (LASSO) Cox regression analysis was performed with “glmnet” package in R to develop a gene signature. The independent variable in the regression was the expression matrix of selected HRGs by univariate analysis, and the response variables were OS and survival status of patients in the training cohort. The optimal penalty parameter (lambda) was determined through 10-fold cross-validation (Tibshirani, 1997). A prognostic gene signature was finally established based on expression levels of the HRGs and the corresponding regression coefficients. Risk score was calculated for each patient as follows: signature risk score = coefficient_1_ × expression level of gene_1_ + coefficient_2_ × expression level of gene_2_ +…+ coefficient_n_ × expression level of gene_n_. According to the optimal cutoff determined by the Youden index method, patients in the training group were divided into high-risk group and low-risk group. Moreover, the training cohort was divided into TCGA and ICGC datasets for internal validation, and external validation was conducted using the GSE14520 dataset.

The receiver operating characteristic (ROC) curve, Kaplan–Meier method, concordance index (C-index), and multivariate Cox regression were utilized to assess the prognostic efficiency of the risk model. Specifically, survival differences between the two risk groups were compared using Kaplan–Meier with log-rank statistical methods using R package “survminer,” and time-dependent ROC curves of 1, 3, and 4 years were performed *via* “survival ROC” package to assess the predictive accuracy of the model in the training and validation cohorts. Additionally, multivariate Cox regression analysis was conducted to determine whether the risk score is an independent risk factor for OS in HCC in the TCGA, ICGC, and GSE14520 datasets, respectively.

Furthermore, we studied the expression profiling of the prognostic gene signature. The mRNA expression of the genes in tumor and non-tumor tissues was analyzed using the Wilcoxon rank-sum test. In addition, the protein expression of the identified genes was explored in the Human Protein Atlas online database (http://www.proteinatlas.org) (Thul and Lindskog, 2018).

### Construction and Assessment of the Nomogram

The independent prognostic factors identified by multivariate Cox regression analysis were applied to construct the nomogram for estimating the OS of 1–3 years in HCC using “rms” package of R. Following that, the calibration curve was used to assess the concordance between actual and predicted survival. The predictive performance of the nomogram was subsequently evaluated using the time-dependent ROC analysis.

### Gene-Set Enrichment Analysis (GSEA)

GSEA (http://software.broadinstitute.org/gsea/index.jsp) was performed based on the training set to identify gene sets that were significantly different between the high- and low-risk groups (Subramanian et al., 2005). After performing 1,000 permutations, gene sets with a nominal *P* < 0.05 were considered statistically enriched.

### Statistical Analysis

The R software (version 3.6.3) was used to present data management, statistical analysis, and data visualization. The Wilcoxon rank-sum test or Kruskal–Wallis test was used to compare differences in clinicopathological features between two groups and among multiple groups. The value of *P* < 0.05 was applied as a threshold of statistical significance.

## Results

### Establishment of Prognostic Gene Signature

**Figure 1:**
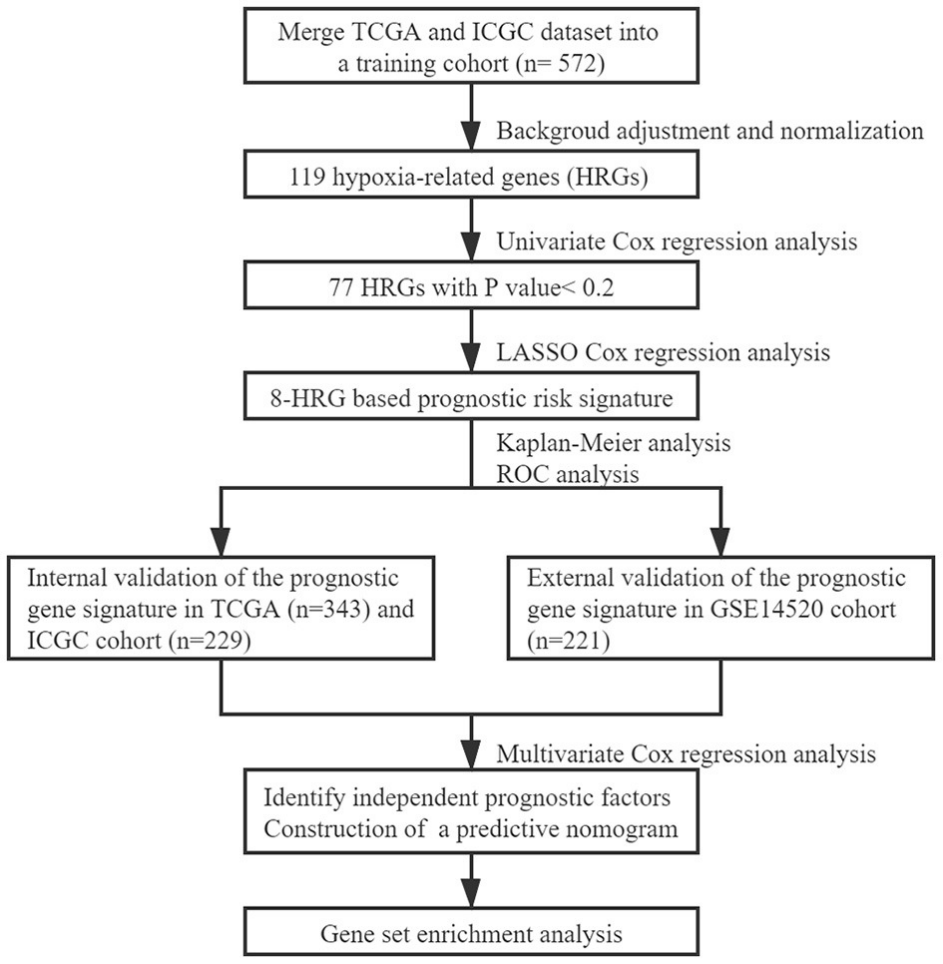
The workflow chart of constructing the prognostic model based on the hypoxia signature.

The workflow chart of the study was displayed in Figure 1. A total of 343 HCC patients from the TCGA cohort, 229 HCC patients from the ICGC cohort, and 221 HCC patients from the GSE14520 cohort were finally enrolled. The representative clinical characteristics of these patients are shown in Table 1.

**Figure 2:**
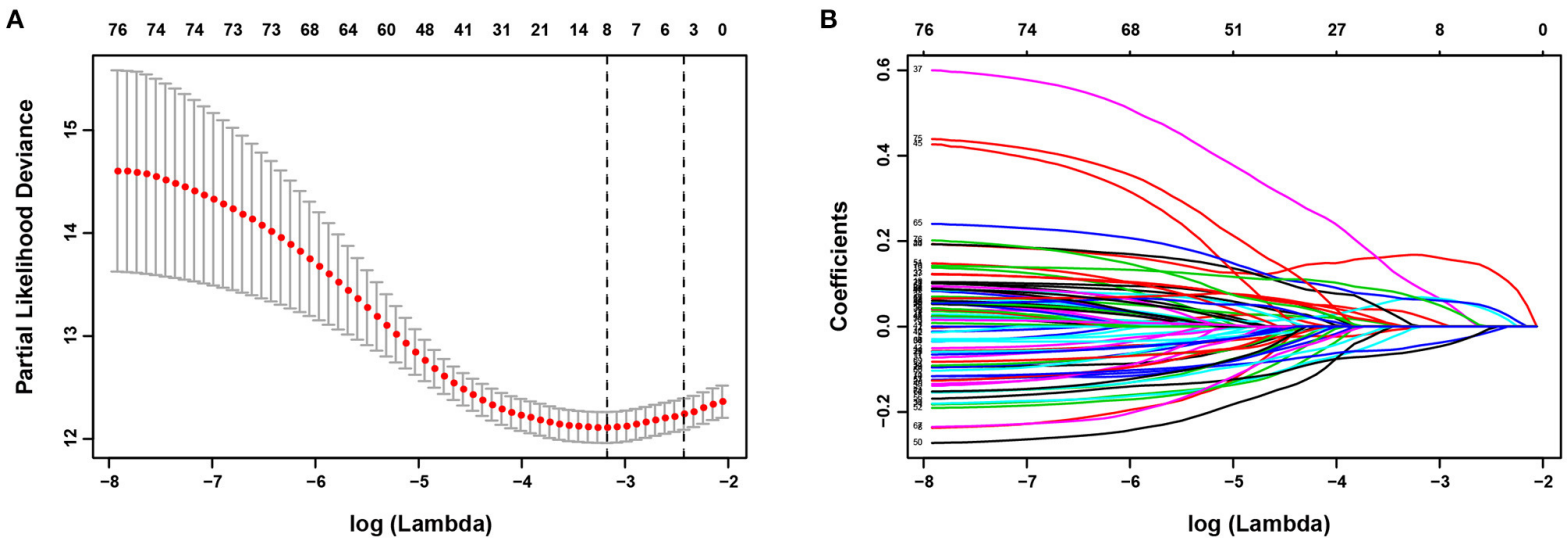
Establishment of prognostic gene signature. **(A)** The optimal log (Lambda) value in the least absolute shrinkage and selection operator (LASSO) model. Red dots and gray lines represent the partial likelihood deviance and error bars, respectively. A vertical line is drawn at the λ value of 0.0418 chosen by 10-fold cross-validation. **(B)** LASSO coefficient profiles of the 77 HRGs.

**Figure 3:**
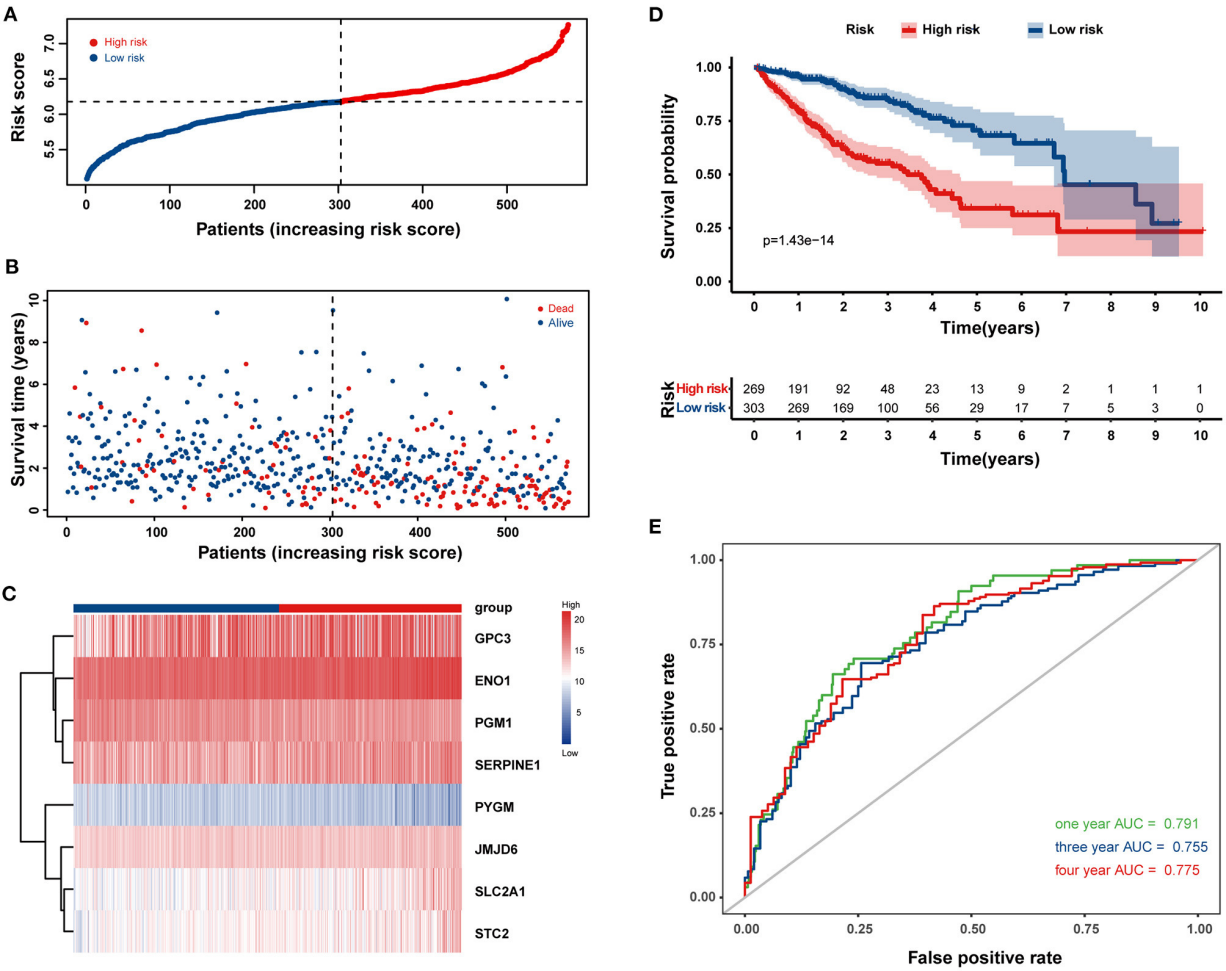
Construction of a prognostic gene signature in the training cohort for overall survival (OS) of HCC patients. The distribution of the risk score **(A)**, survival time and life status **(B)**, and the eight-HRG expression profiles **(C)** for the 572 HCC patients. **(D)** Kaplan–Meier curves comparing OS of HCC patients in the high- and low-risk groups. **(E)** The time-dependent receiver operating characteristic (ROC) curves of the eight-HRG signature for predicting OS at 1, 3, and 4 years.

Univariate Cox regression analysis identified 77 HRGs with a *P* < 0.2. The expression levels of eight HRGs and the corresponding coefficients based on LASSO Cox regression analysis were used to construct a prognostic gene signature (Figure 2). The risk score for predicting OS was calculated as follows: risk score = [Enolase 1 (ENO1) × (0.1676)] + [Glypican 3 (GPC3) × (0.0688)] + [Jumonji domain containing 6 (JMJD6) × (0.0875)] + [Phosphoglucomutase 1 (PGM1) × (−0.0549)] + [Muscle glycogen phosphorylase (PYGM) × (−0.0437)] + [Serpin peptidase inhibitor type 1 (SERPINE1) × (0.0228)] + [Solute carrier family 2 member 1 (SLC2A1) × (0.0633)] + [Stanniocalcin 2 (STC2) × (0.0638)]. Furthermore, the risk score was generated for each patient in the training cohort. According to the optimal risk score cutoff value, patients were divided into high-risk group (*n* = 269) and low-risk group (*n* = 303). And the distributions of the risk scores, OS time, and heat map in the training cohort are shown in Figures 3A–C. In addition, Kaplan–Meier curves showed that patients in the low-risk group had a significantly better OS than those in the high-risk group in the training dataset (log-rank *P* < 0.001; Figure 3D). The areas under curves (AUC) of the ROC curves for predicting 1-, 3-, and 4-year OS of HCC in the training cohort were 0.791, 0.755, and 0.775, respectively (Figure 3E). Moreover, the C-index of the HRG-based prognostic risk model for OS prediction was 0.751 for the training cohort.

### Internal Validation of the Prognostic Gene Signature

**Figure 4:**
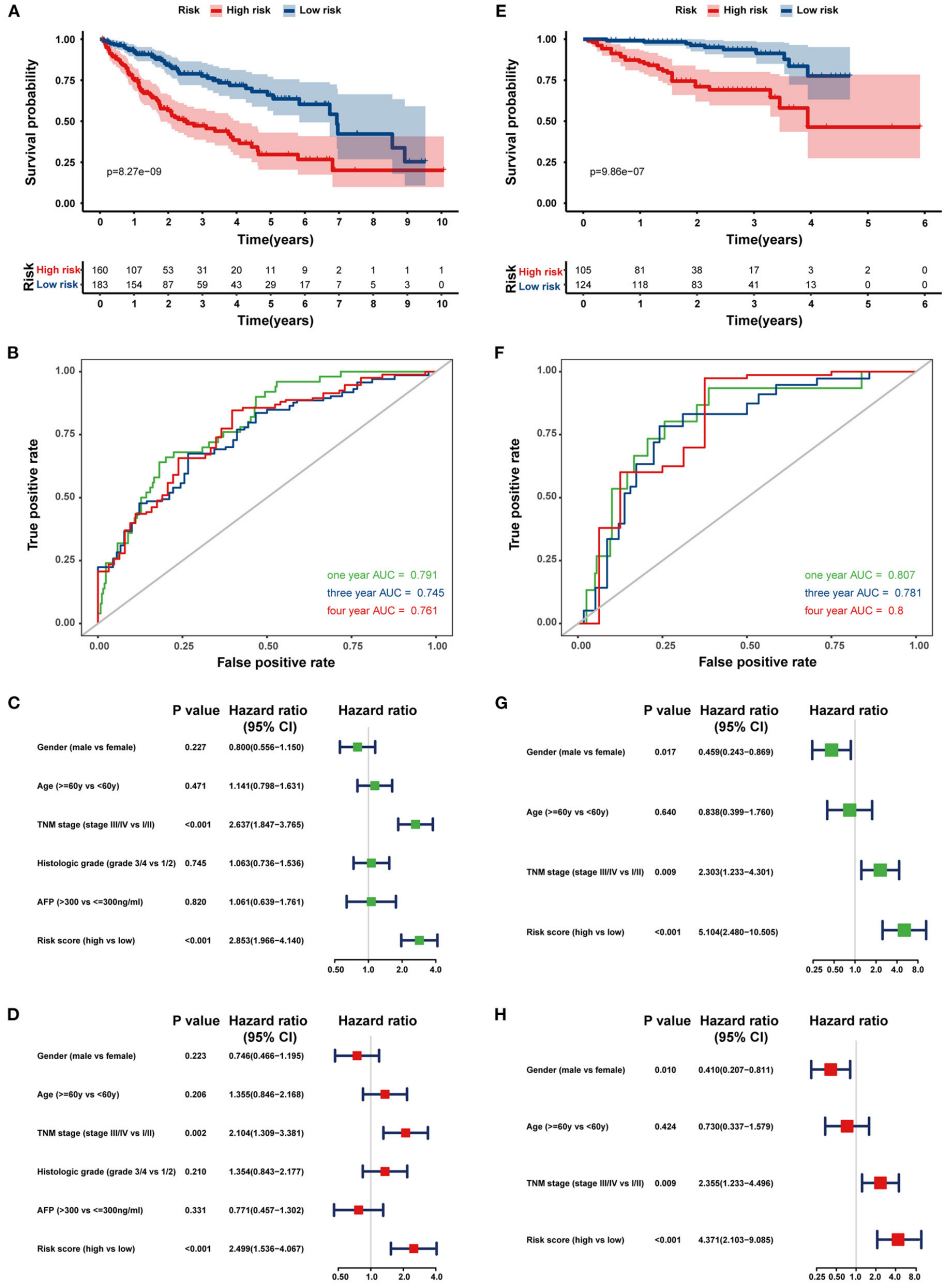
Internal validation of the prognostic gene signature in the TCGA and ICGC cohorts. Kaplan–Meier curves and time-dependent ROC curves at 1, 3, and 4 years of predicting the OS of HCC patients in the TCGA **(A,B)** and ICGC cohorts **(E,F)**. Univariate (Green) and multivariate (Red) Cox regression analyses of the association between clinicopathological parameters and hypoxia risk signature regarding the OS of HCC patients in the TCGA **(C,D)** and ICGC cohort **(G,H)**.

Next, the 8-HRG-based signature was internally validated in the TCGA and ICGC cohorts. In the TCGA cohort, patients in the high-risk group had a significantly worse OS than those in the low-risk group (Figure 4A). The AUCs of the risk score for predicting 1-, 3-, and 4-year OS were 0.791, 0.745, and 0.761, respectively (Figure 4B). In accordance with the results above, high-risk patients showed a lower OS rate in the ICGC cohort (Figure 4E). The AUCs for 1-, 3-, and 4-year OS were 0.807, 0.781, and 0.800, respectively (Figure 4F). Univariate and multivariate Cox regression analyses demonstrated that tumor-node-metastasis (TNM) stage and the risk score were independent prognostic factors for OS in both TCGA and ICGC cohorts (Figures 4C,D,G,H).

### External Validation of the Prognostic Gene Signature

**Figure 5:**
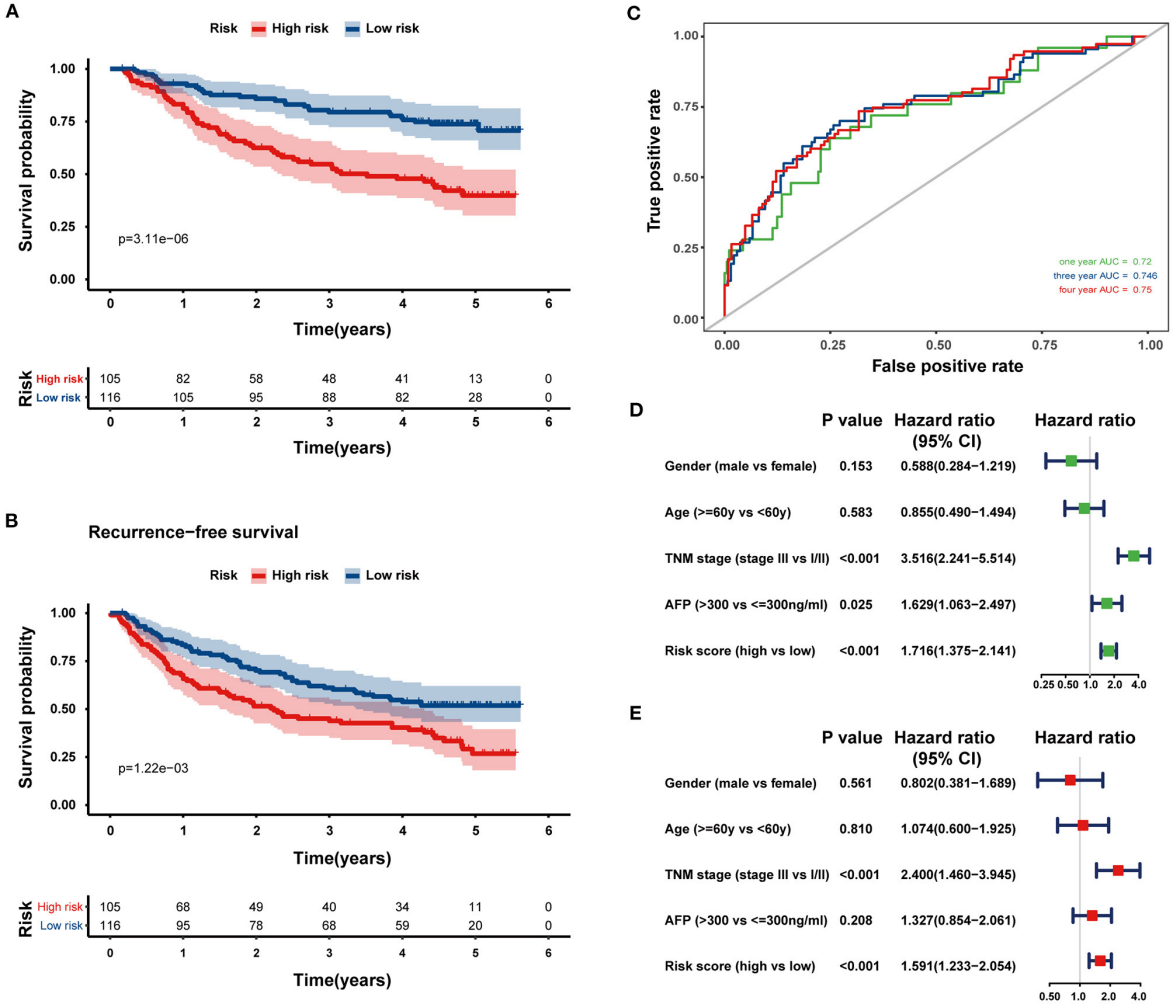
External validation of the prognostic gene signature in the GSE14520 dataset. Kaplan–Meier curves comparing OS **(A)** and recurrence-free survival **(B)** curves of HCC patients with high or low hypoxia risk. **(C)** The ROC curves of the eight-HRG signature for predicting OS at 1, 3, and 4 years in the GSE14520 cohort. Univariate **(D)** and multivariate **(E)** Cox regression analyses of clinicopathological parameters and hypoxia risk signature of HCC patients in the GSE14520 cohort.

To confirm the predictive ability of the eight-HRG-based signature, an external validation analysis was conducted in the GSE14520 cohort. The risk score regarding each patient was calculated according to the same formula as that from the training dataset, and patients were also stratified into two risk groups, including 105 patients in the high-risk group and 116 patients in the low-risk group. Consistent with the results in the training cohort, patients in the high-risk group showed a significantly lower OS (log-rank *P* < 0.001; Figure 5A) and recurrence-free survival (log-rank *P* = 0.001, Figure 5B) in comparison with those in the low-risk group. While in GSE14520 cohort, the AUC values for the risk scores predicting 1-, 3-, and 4-year OS were 0.720, 0.746, and 0.750, respectively (Figure 5C). As indicated by univariate and multivariate Cox regression analysis, the risk score could independently predict OS (Figures 5D,E). The ROC comparisons between hypoxia-related signature and other prognostic models reported in previous studies are shown in Supplementary Figure 1.

### Expression Profiling of Identified Eight HRGs

**Figure 6:**
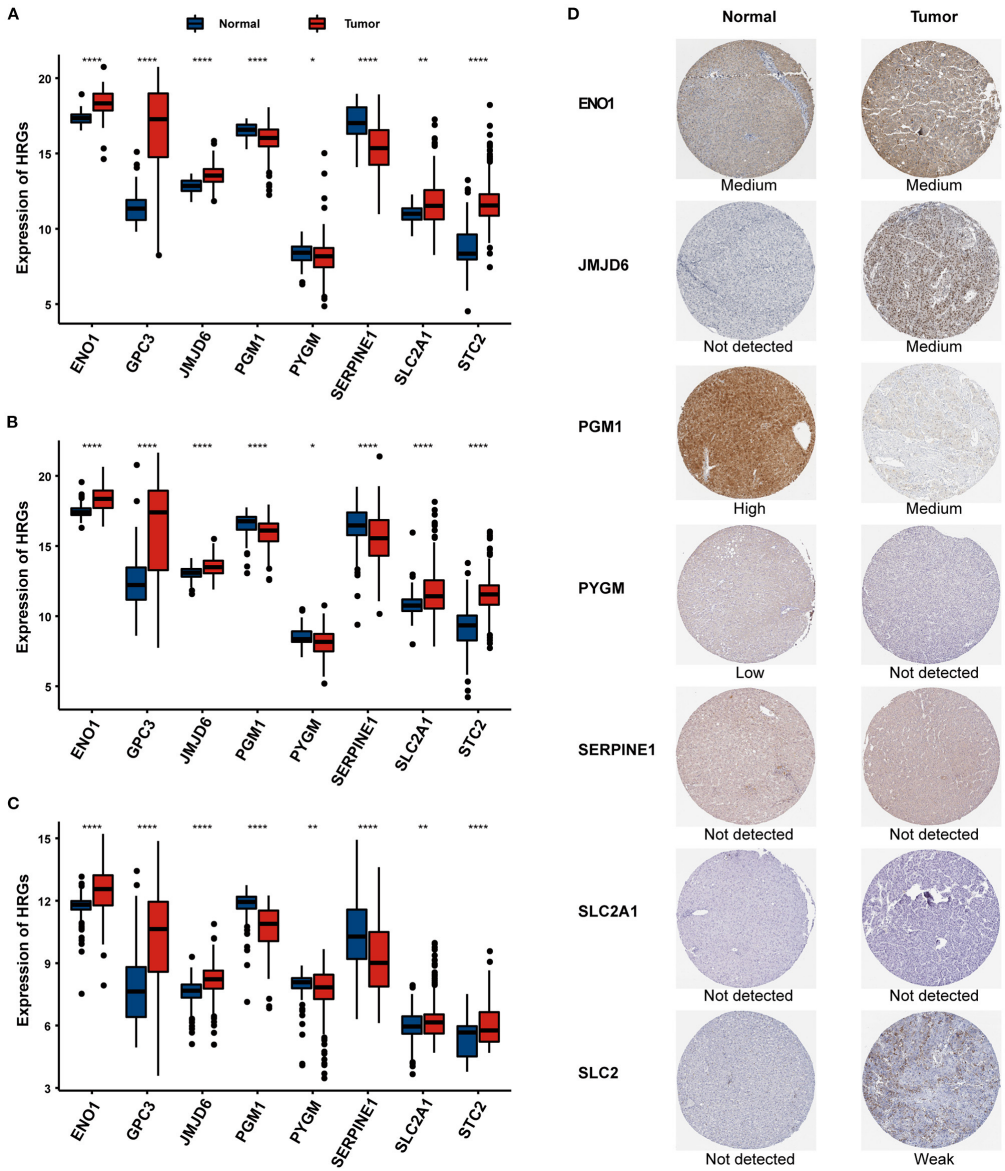
The expression of the eight HRGs in HCC. The RNA-seq expression of the eight genes in the TCGA **(A)**, ICGC **(B)**, and GSE14520 **(C)** cohorts. **(D)** The expression of proteins encoded by the representative genes in normal and HCC tissues using specimens from the Human Protein Profiles. *P < 0.05, **P < 0.01, ***P < 0.001.

The mRNA expression patterns of the selected eight HRGs between HCC and normal tissues in the three independent cohorts are shown in Figures 6A–C, revealing that PGM1, PYGM, and SERPINE1 were significantly downregulated in HCC tissues while the other five genes were significantly upregulated relative to non-tumor tissues. We further explored the protein expression encoded by these genes. As shown in Figure 6D, ENO1 and JMJD6 were moderately positive while SLC2 was weakly positive in HCC tissues when compared with corresponding expression levels in non-tumor tissues. In contrast, PGM1 and PYGM showed strong and low positive in normal liver tissues, respectively.

### Association Between the Risk Signature and Clinicopathological Characteristics

The association between the HRG-based risk signature and clinicopathological characteristics was subsequently explored. No significant differences were observed between males and females or various age groups in either the TCGA, ICGC, or GSE14520 dataset. In comparison with grades 1–2, patients with grades 3–4 had significantly higher risk scores in the TCGA cohort (*P* < 0.001; Supplementary Figure 2A). Moreover, HCC patients with higher TNM stages had obviously higher risk scores in the TCGA (Supplementary Figure 2B), ICGC (Supplementary Figure 2D), and GSE14520 (Supplementary Figure 2E) cohorts (all *P* < 0.01). In addition, as tumor stage increased, risk scores showed a significantly increasing trend in the TCGA cohort (*P* < 0.01; Supplementary Figure 2C). Similarly, risk scores were significantly higher in patients with tumor size ≤ 5 cm than those with tumor size >5 cm in the GSE14520 cohort (*P* = 0.001, Supplementary Figure 2F).

### Construction and Assessment of the Predictive Nomogram

**Figure 7:**
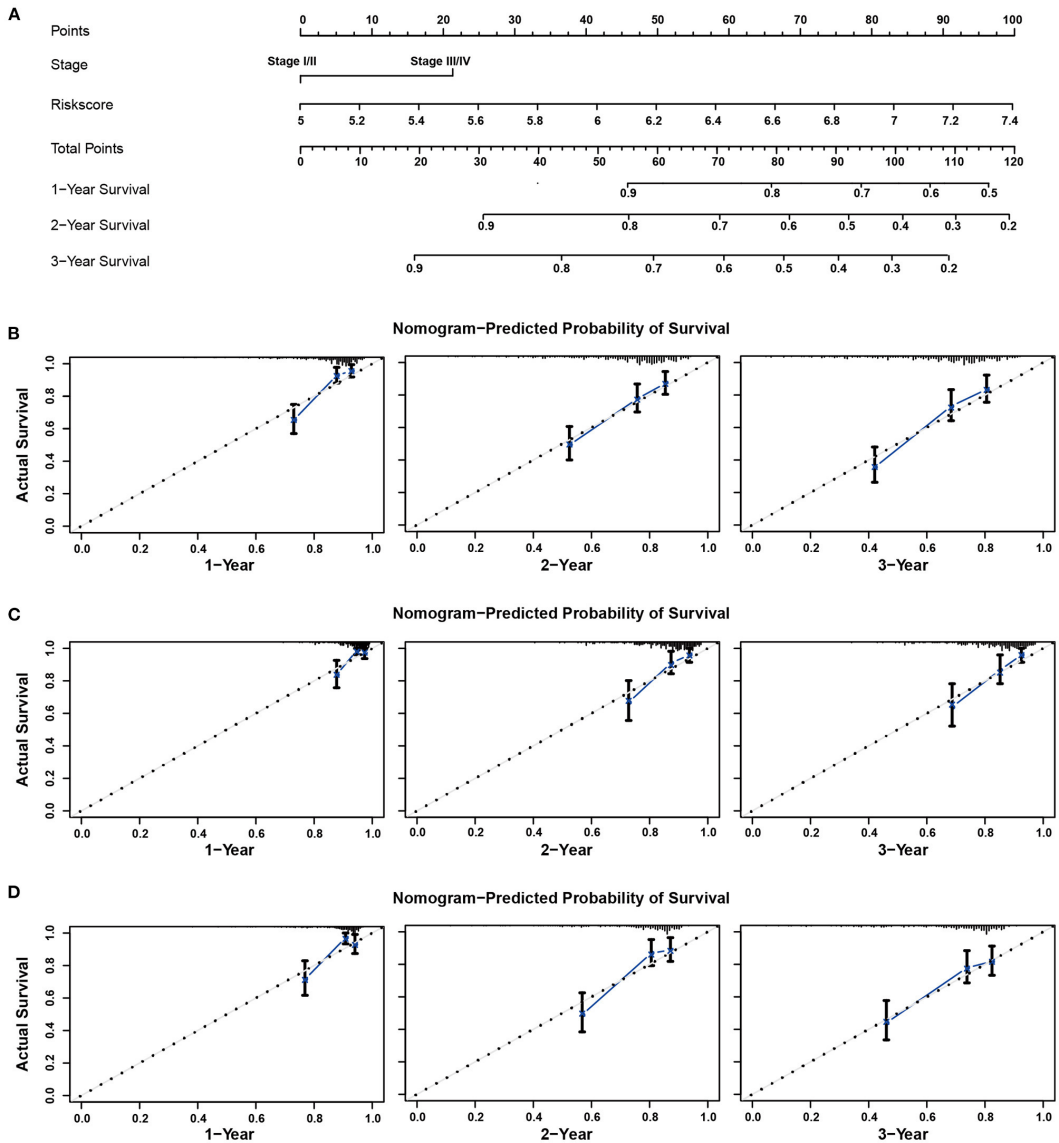
Nomogram to predict overall survival for patients with HCC. **(A)** The prognostic nomogram for predicting the survival probability of HCC patients based on the TCGA cohort. The calibration curves of the nomogram for predicting OS at 1–3 years in the TCGA **(B)**, ICGC **(C)**, and GSE14520 **(D)** cohorts.

The risk scores and TNM stage of patients in the training cohort were used for constructing a nomogram for OS predication. One, two, and three-year calibration curves are presented in Figure 7A. In the TCGA and ICGC cohorts, the calibration plots demonstrated an excellent agreement between the predicted and actual OS (TCGA, Figure 7B; ICGC, Figure 7C). In addition, the calibration plots showed a favorable prediction ability for the survival rates in the GSE14520 cohort (Figure 7D). The C-index for the nomogram and TNM stage was 0.774 and 0.639, respectively. As shown in Supplementary Figure 3, the AUCs of the nomogram for predicting 1-, 2-, and 3-year OS were 0.821, 0.772, and 0.785, respectively. Compared with risk score and TNM stage alone, the combination of risk score and TNM stage showed larger AUCs for 1-, 2-, and 3-year OS.

### Gene-Set Enrichment Analysis

**Figure 8:**
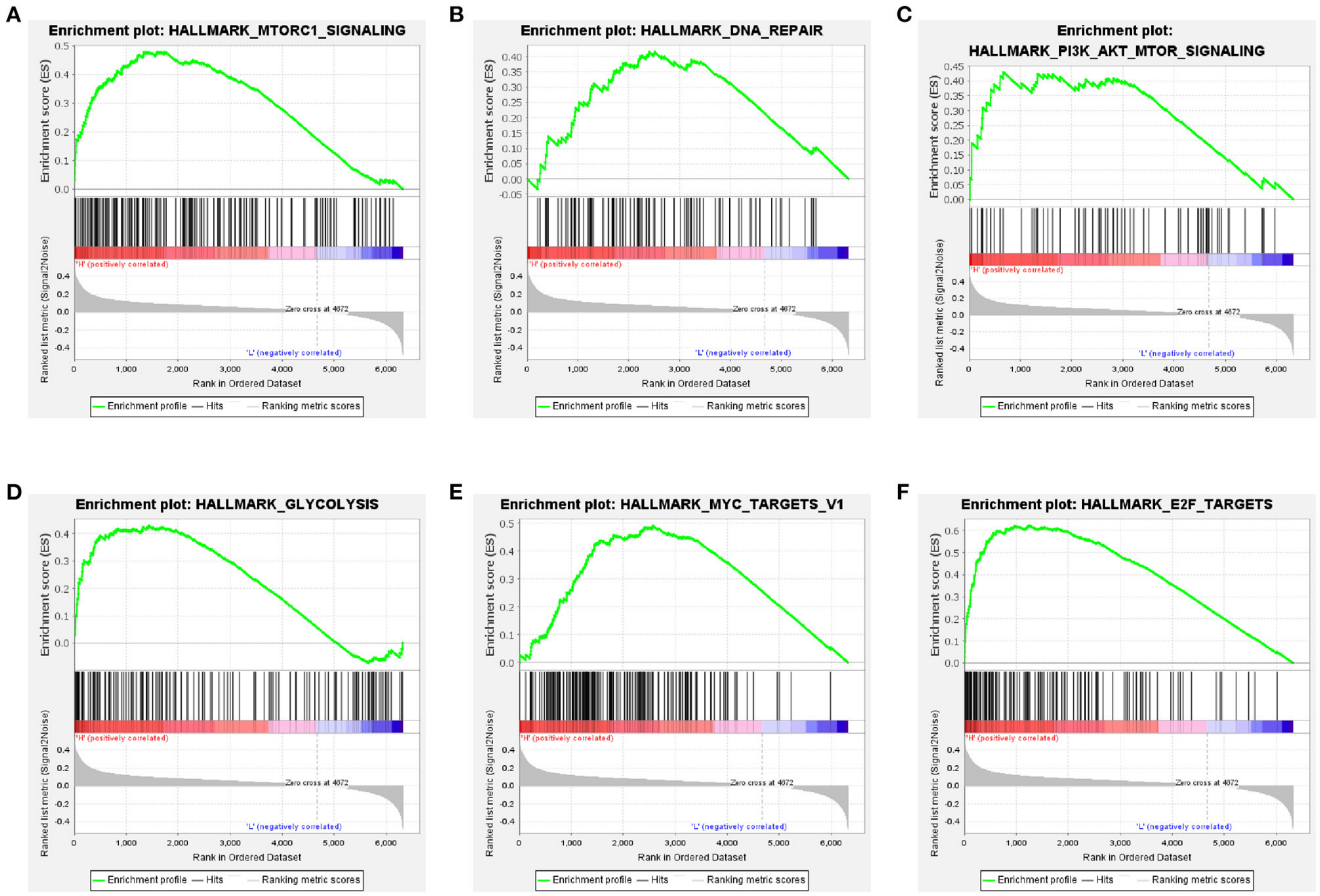
Gene-set enrichment analyses between high- and low-risk groups based on the training cohort. Genes in the high-risk group were enriched for hallmarks of the mTORC1 signaling pathway **(A)**, DNA repair **(B)**, PI3K/Akt/mTOR signaling pathway **(C)**, glycolysis **(D)**, MYC targets V1 **(E)**, and E2F targets **(F)**.

GSEA analysis revealed that the high-risk group was significantly associated with mammalian target of rapamycin (mTOR) complex 1 signaling pathway, DNA repair, phosphatidylinositol 3-kinase (PI3K)/protein kinase B (Akt)/mTOR signaling pathway, and glycolysis (Figures 8A–D). Furthermore, oncological signatures, including myelocytomatosis oncogene (MYC) and E2F transcription factor (E2F), were also significantly enriched in the high-risk group (Figures 8E,F).

## Discussion

Recently, with the vigorous development of medical technology, the needs of patients with tumors are not limited to disease diagnosis or conventional treatment. The concept of precision medicine is requesting molecular diagnosis and personalized therapeutic method according to the differential expression of genetic level. Hence, a novel predictive signature based on eight HRGs for HCC has been established in this study and validated in an independent cohort to meet the urgent need of effective prognostic biomarkers reflecting risk stratification and survival outcomes of HCC patients. The results suggest that the gene signature can successfully predict the OS of patients with HCC.

In general, the HIF is a key regulator of the cellular hypoxia response that plays crucial roles in tumor resistance to different treatment modalities and poor prognosis in HCC (Burroughs et al., 2013; Fallah and Rini, 2019). There are more than a hundred genes regulated by HIF in an induced or repressed method (Manalo et al., 2005), which primarily leads to a reprogramming of glucose metabolism and regulates epithelial–mesenchymal transition (Denko, 2008; Taniguchi et al., 2013). Significantly, the glycolytic phenotype was often associated with a higher level of HIF-1 in tumor cells, while the hybrid metabolic phenotype combined with glycolysis and oxidative phosphorylation was characterized by the intermediate level of HIF-1 (Dupuy et al., 2015; Jia et al., 2019). The modulation of metabolic pathways in tumor cells could be understood by the gene-regulated metabolic plasticity. In the present study, LASSO Cox regression analysis helped select the eight HRGs (ENO1, GPC3, JMJD6, PGM1, PYGM, SERPINE1, SLC2A1, and STC2) from a total of 77 genes to develop the hypoxia gene signature, which was an independent prognostic factor for HCC patients (Table 2). These genes could be roughly classified into four categories: glucose metabolism (SLC2A1, ENO1), glycogen metabolism (PGM1, PYGM), malignant biological behavior (JMJD6, GPC3, STC2), and coagulation function (SERPINE1). In HCC cells, glucose addiction drove an increasing glucose flex into the cytoplasm transported by SLC2A1 (known as GLUT1) in response to hypoxia, whose higher expression level provided the primary motivation for the metabolism reprogramming (Nagarajan et al., 2017), such as aerobic glycolysis. Afterward, the catalytic activation of ENO1 was also enhanced *via* HIF, promoting survival of cancer cells in the hypoxia area by the modulating glycolytic metabolism (Yu et al., 2018). Previous studies have shown that not all the HIF-1 signature genes are expressed higher in the tumor tissues than these in the normal tissues (Jia et al., 2019). In the process of glycogen metabolism, the low expression of PGM1 hindered the glycogen synthesis pathway of tumor cells, making glucose more used in the glycolysis process, thereby promoting tumor cell proliferation and the malignant progression of HCC (Jin et al., 2018). Additionally, as an important enzyme in the first step of glycogenolysis (Favaro et al., 2012), PYGM was found to be less expressed in tumor tissues than that in normal tissues, which made an impact on the survival of breast cancer patients. These data above implied the potential relationship between the decreasing expression of PGM1 and PYGM and the poor prognosis of HCC patients. Apart from the influence on glucose metabolism, the gene signature also included vital indicators associated with liver function, cell proliferation, gene transcription, and diagnosis. Of note, for a histone arginine demethylase or lysyl oxidase to target histones, overexpression of JDJM6 was related to the poor OS in HCC by modulating RNA splicing (Wan et al., 2019). Significantly, under the hypoxic state, increase in SERPINE1 could lead to the thrombosis and consequent inhibition of fibrinolysis through an HIF-induced method (Sanagawa et al., 2016), which supplemented another aspect reflecting the liver function and coagulation function. Moreover, a gene for modern detection and diagnosis of HCC, GPC3, was taken into consideration as a marker in liver biopsies. GPC3 was a cell surface heparan sulfate proteoglycan that could enhance HCC cell migration *via* upregulating the expression under hypoxia states and interact with epithelial–mesenchymal transition-associated targets (Meng et al., 2021). Surprisingly, a glycosylated peptide hormone responsible for the balance of calcium and phosphorus, STC2, was found to have an impact on proliferation, migration, and association with poor outcomes in HCC (Wang et al., 2019). STC2 and SLC2A1 were both involved in biological behaviors of many cancers, especially in HCC, through the modulation of mTOR signaling pathways (Wei et al., 2017; Wu et al., 2017). Also, one of the reasons for understanding this reprogramming of metabolism was the realization of the PI3K/AKT/mTOR complex 1 signaling pathway that was frequently activated in cancer cells (Hay, 2016). Consistently, our findings of GSEA also indicated the participation of the mTOR signaling pathway and further detected the involvement of DNA repair and glycolysis in HCC.

**Table 2:** The function and mechanism of 8 hypoxia-related genes (HRGs) in hepatocellular carcinoma (HCC).

**HRGs**	**Full name**	**Description**	**Mechanism**	**References**
ENO1	Enolase 1	A key glycolytic enzyme	Promote survival of cancer cells in the hypoxia area by the modulating glycolytic metabolism	Yu et al., 2018
GPC3	Glypican 3	A cell surface heparan sulfate proteoglycan	Enhance HCC cell migration via upregulating expression and interacting with epithelial-mesenchymal transition -associated targets	Meng et al., 2021
JMJD6	Jumonji domain containing 6	A histone arginine demethylase or lysyloxidase to target histones	Influence the overall survival in HCC by modulating RNA splicing	Wan et al., 2019
PGM1	Phosphoglucomutase 1	An evolutionary conserved enzyme of the ubiquitous and ancient α-D-phosphohexomutases	Hinder the glycogen synthesis pathway of tumor cells and promote proliferation and the malignant progression of HCC	Jin et al., 2018
PYGM	Muscle glycogen phosphorylase	An important enzyme in the first step of glycogenolysis	Have impact on the survival of cancer with low expression in tumor cells	Favaro et al., 2012
SERPINE1	Serpin peptidase inhibitor type 1	A fibrinolysis-related indicator	Lead to the thrombosis and consequent inhibition of fibrinolysis through a HIF-induced method	Sanagawa et al., 2016
SLC2A1	Solute carrier family 2 member 1	Facilitative glucose transporter	Provide the primary motivation for the metabolism reprograming	Nagarajan et al., 2017
STC2	Stanniocalcin 2	A human glycoprotein hormone	Responsible for the balance of calcium and phosphorus	Wang et al., 2019

To date, several studies have revealed the role of gene signature in predicting survival outcomes of HCC. Specifically, Wang et al. (2020) identified a five immune-related gene signature, Liu et al. (2020) developed a four-gene metabolic signature, and Xu et al. (2021) generated an eight autophagy-related gene signature. However, our eight-HRG signature had a higher AUC in survival prediction than the above three gene signatures. On the other hand, we generated a nomogram incorporating the TNM stage and hypoxia gene signature to predict individuals' prognosis. In accordance with previous results (Ouyang et al., 2020; Zhang et al., 2020), the combination of the gene signature and TNM staging system achieved the better prognostic prediction performance than using clinical or genetic features alone in HCC, which might facilitate selection of individualized management in the clinical setting. Recently, Zhang et al. (2020) also developed a prognostic model based on three hypoxia gene signatures and further constructed a nomogram predicting OS for HCC patients, and the AUCs of 1- and 3 year-OS were 0.672 and 0.684, respectively. In addition, the AUC values of nomogram integrating autophagy-related signature, cirrhosis, and tumor size in predicting 3-year OS of HCC were 0.638 (Fang and Chen, 2020). At the expense of the acquisition of the expression of more genes in tumor tissues, our nomogram had an excellent performance in predicting survival of HCC patients. There was no doubt that requirement of more gene expression levels complicated the clinical application of the predictive models. However, as the advancement of molecular detection, the nomogram based on gene signature may be routinely applied in the future.

Altogether, we identified eight HRGs and integrated them into a gene signature. This signature could stratify patients into different risk groups in clinical assessment through the gene expression levels, which provided a potential option for personalized treatments. Moreover, a nomogram based on the combination of the gene signature derived from the mRNA expression of eight genes and conventional TNM stage showed a good performance in predicting survival outcomes of HCC patients. According to the nomogram, the total point could be calculated for each patient. Then, the specific 1-, 2-, and 3-year OS could be concluded based on the corresponding point, which may assist physicians to predict prognosis of HCC patients and determine individual treatment options for different patients. Nevertheless, there are some limitations to this study. First, the functions of these genes and the underlying mechanisms need to be further analyzed and verified by experiments. Second, our findings are based on public datasets, which needs further validation in a prospective study.

## Conclusions

The present study established a prognostic gene signature of eight HRGs, which was proven to be valuable in reflecting risk classification and might provide therapeutic targets for patients with HCC. A nomogram combining the eight-HRG signature and TNM stage was developed for 1–3-year OS prediction of individual HCC patients. These findings suggest that the eight-hypoxia gene signature may facilitate individualized management for HCC patients in the clinical practice.

## Data Availability Statement

The original contributions presented in the study are included in the article/Supplementary Material, further inquiries can be directed to the corresponding author/s.

## Author Contributions

MJ: conception, design, and critical revision of the manuscript. QL and LJ: analysis and interpretation of data. QL: drafting the manuscript. All authors read and approved the manuscript.

## Conflict of Interest

The authors declare that the research was conducted in the absence of any commercial or financial relationships that could be construed as a potential conflict of interest.

## Supplementary Material

Supplementary Figure 1 The ROC comparation between hypoxia-related signature and other prognostic models in the TCGA **(A,B)** and GSE14520 cohorts **(C,D)**.

Supplementary Figure 2 The clinicopathological significance of prognostic model in HCC. Distribution of the risk scores in different groups classified by **(A)** tumor grades, **(B)** TNM stages, and **(C)** T stages in the TCGA cohort, TNM stages in the ICGC cohort **(D)**, TNM stages **(E)**, and tumor size **(F)** in the GSE14520 cohort.

Supplementary Figure 3 The time-dependent ROC curves of the nomogram comparing 1- **(A)**, 2- **(B)**, and 3-year OS **(C)** in the training cohort.
