# Supplementary figures and images for: Novel Hypoxia-Related Gene Signature for Risk Stratification and Prognosis in Hepatocellular Carcinoma
Source: Front Genet. 2021 Jun 14;12:613890. doi: 10.3389/fgene.2021.613890 (PMC8236897; doi:10.3389/fgene.2021.613890)

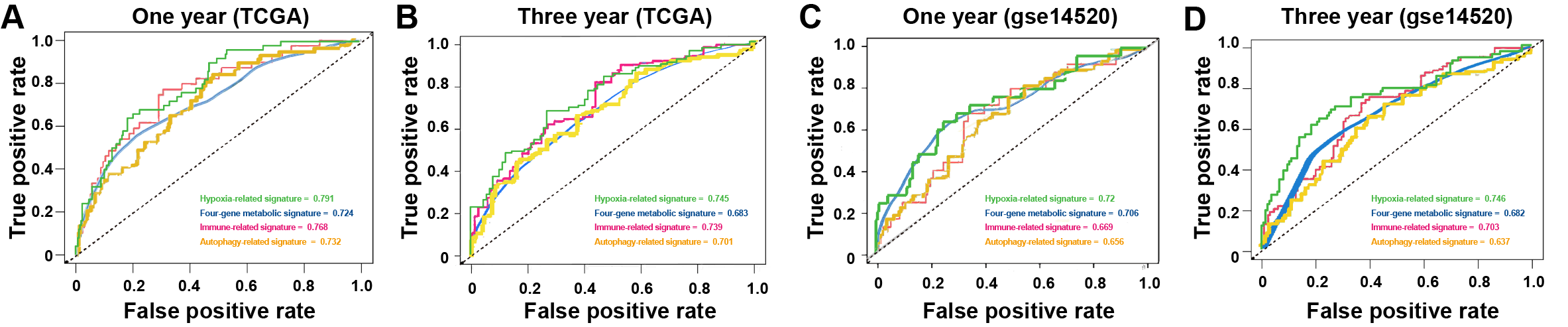

Supplement: Supplementary Figure 1 — The ROC comparation between hypoxia-related signature and other prognostic models in the TCGA (A,B) and GSE14520 cohorts (C,D). [file Image_1.TIF]

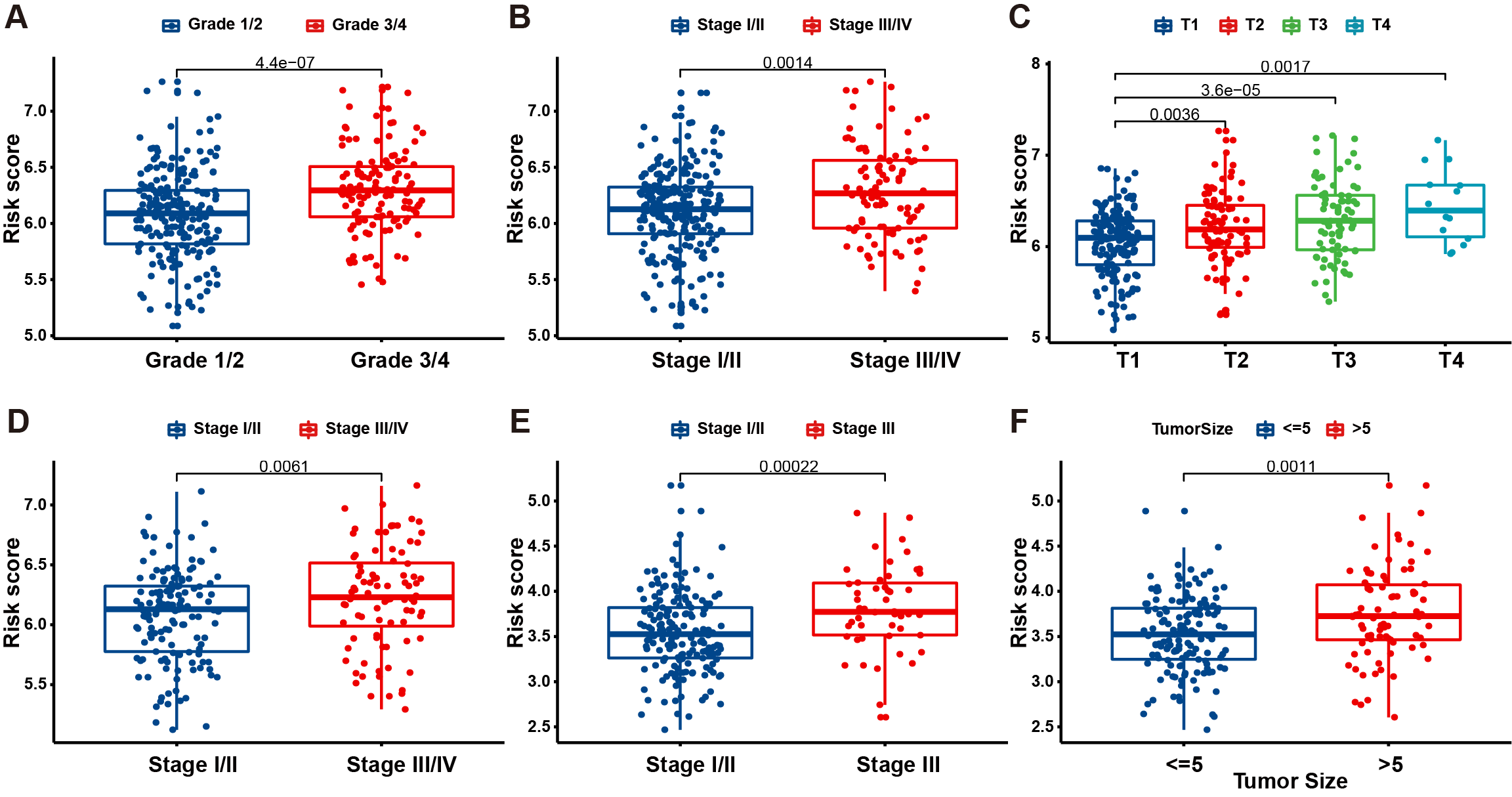

Supplement: Supplementary Figure 2 — The clinicopathological significance of prognostic model in HCC. Distribution of the risk scores in different groups classified by (A) tumor grades, (B) TNM stages, and (C) T stages in the TCGA cohort, TNM stages in the ICGC cohort (D), TNM stages (E), and tumor size (F) in the GSE14520 cohort. [file Image_2.TIF]

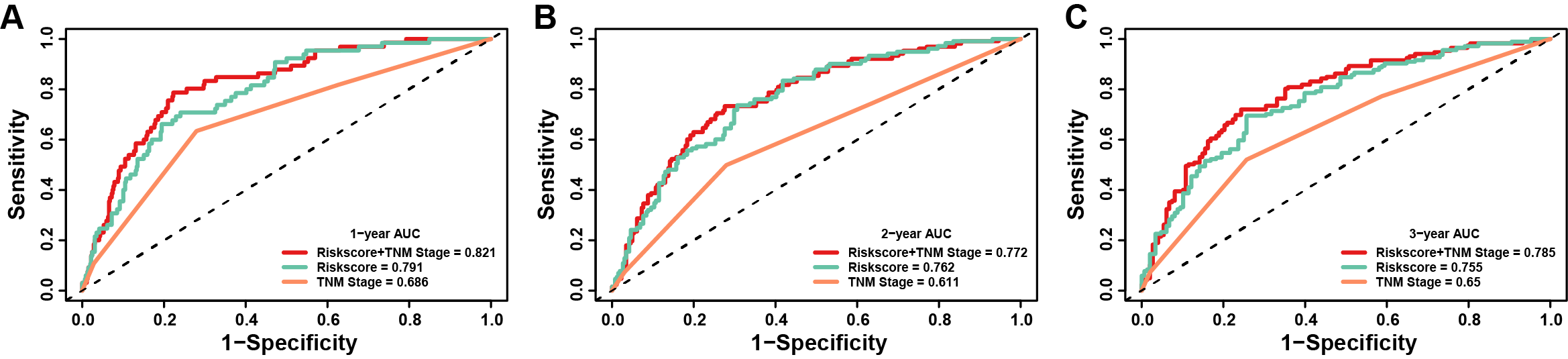

Supplement: Supplementary Figure 3 — The time-dependent ROC curves of the nomogram comparing 1- (A), 2- (B), and 3-year OS (C) in the training cohort. [file Image_3.TIF]
